# Surgical Management of Omphalocele With Concurrent Ileal Atresia: A Case Report

**DOI:** 10.7759/cureus.59147

**Published:** 2024-04-27

**Authors:** Sai Goutham Rekavari, Kiran Khedkar, Chanrashekhar Mahakalkar, Sanjeev Gianchandani

**Affiliations:** 1 General Surgery, Jawaharlal Nehru Medical College, Datta Meghe Institute of Higher Education and Research, Wardha, IND; 2 Pediatric Surgery, Jawaharlal Nehru Medical College, Datta Meghe Institute of Higher Education and Research, Nagpur, IND

**Keywords:** multidisciplinary care, surgical management, congenital anomalies, neonatal surgery, ileal atresia, omphalocele

## Abstract

Omphalocele, a congenital anomaly characterized by the protrusion of abdominal viscera through the umbilical ring, often presents challenges in surgical management, especially when concurrent with other anomalies such as intestinal atresia. We presented a case of a female infant weighing 2.6 kg born with omphalocele and concurrent ileal atresia. The child was successfully managed through prompt surgical intervention. Preoperative investigations revealed signs suggestive of intestinal obstruction, necessitating immediate surgical exploration. Intraoperatively, meticulous reduction of the omphalocele sac and resection of the atretic segment were performed. Postoperative care in the neonatal intensive care unit ensured optimal recovery. This case underscored the importance of timely intervention and multidisciplinary collaboration in managing complex congenital anomalies in neonates.

## Introduction

Omphalocele is a rare congenital anomaly characterized by the protrusion of abdominal viscera through the umbilical ring, typically covered by a sac composed of peritoneum and amnion. During embryonic development, the abdominal wall fails to close properly, causing this condition [1]. The exact etiology of omphalocele remains unclear; however, it is believed to involve both genetic and environmental factors [2]. Omphalocele is often associated with other congenital anomalies, including cardiac defects, neural tube defects, and gastrointestinal malformations [3]. Intestinal atresia is a common coexisting anomaly in infants with omphalocele. It is characterized by the complete or partial obstruction of the intestinal lumen due to a congenital absence or narrowing of a segment of the intestine [4]. The etiology of intestinal atresia is multifactorial and includes vascular accidents, intrauterine vascular disruptions, and genetic predispositions [5].

Managing omphalocele with concurrent intestinal atresia requires a multidisciplinary approach involving neonatologists, pediatric surgeons, and other specialists. Timely diagnosis and surgical intervention are essential to prevent complications such as intestinal obstruction, sepsis, and nutritional deficiencies [6]. Advancements in neonatal intensive care, surgical techniques, and perioperative management have improved outcomes for infants with omphalocele and intestinal atresia. However, challenges remain in optimizing long-term prognosis and minimizing morbidity in this vulnerable population [7].

## Case presentation

A 2.6-kg female infant was delivered to a mother with a gravidity of two, a parity of one, and a history of one living child at full term via normal vaginal delivery on March 24, 2023, at 11:30 p.m. at Government Hospital. The infant cried immediately after birth and was promptly placed with the mother. The neonate did not pass stools after birth or tolerate tube feeding, which suggested intestinal obstruction. The infant was diagnosed with a congenital disability known as omphalocele and was subsequently referred to a tertiary-care hospital for further management.

Upon admission, the infant was maintained on nasogastric tube feeding and initiated on intravenous fluids, ampicillin, and gentamicin. Preoperative investigations, including complete blood count, kidney function tests, liver function tests, and coagulation profile, were performed, all of which yielded results within normal limits: hemoglobin 19.1 g/dL, total leukocyte count 19,800/mm³, and platelet count 181,000/mm³.

**Figure 1:**
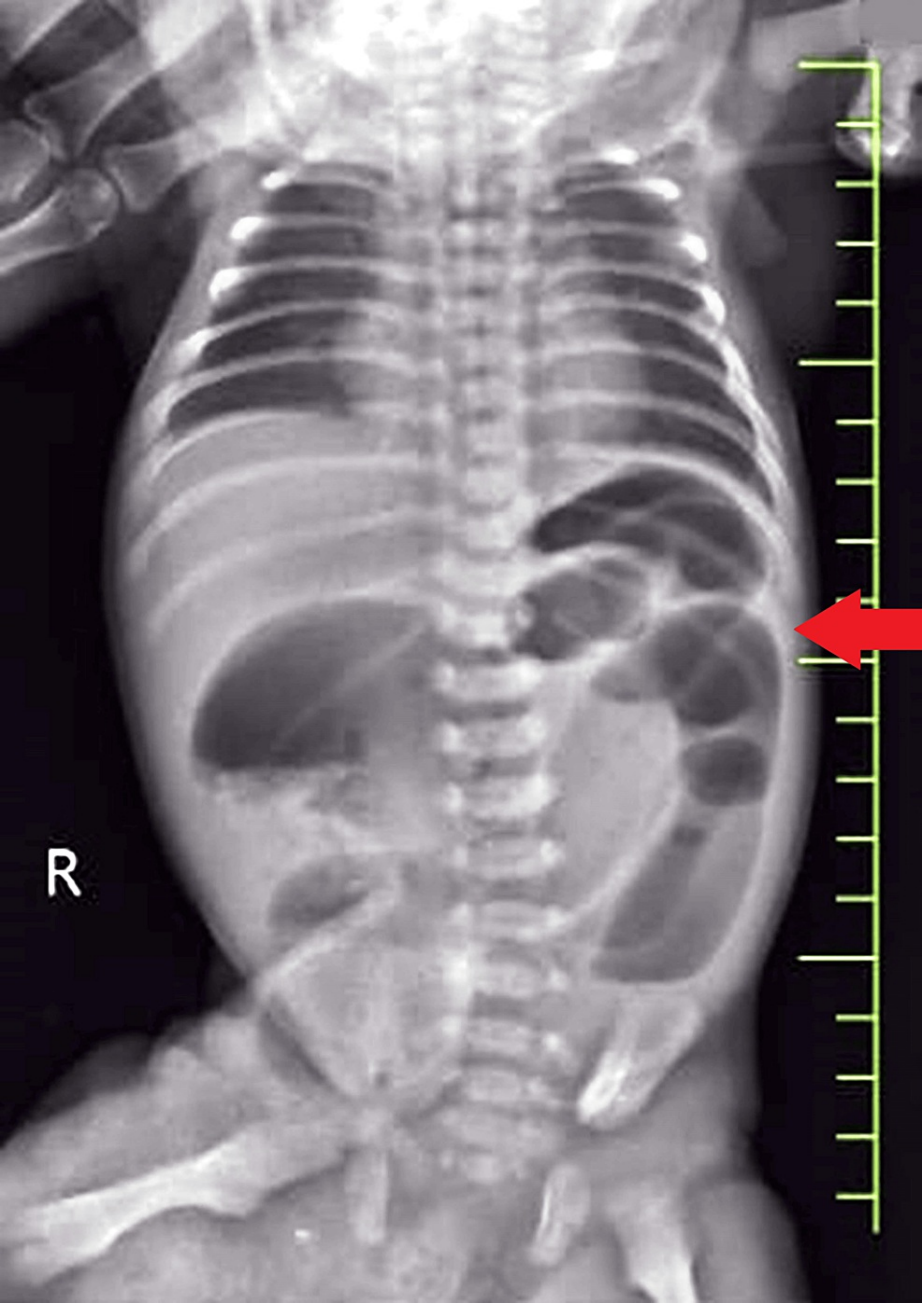
X-ray revealed signs suggestive of intestinal obstruction (red arrow)

**Figure 2:**
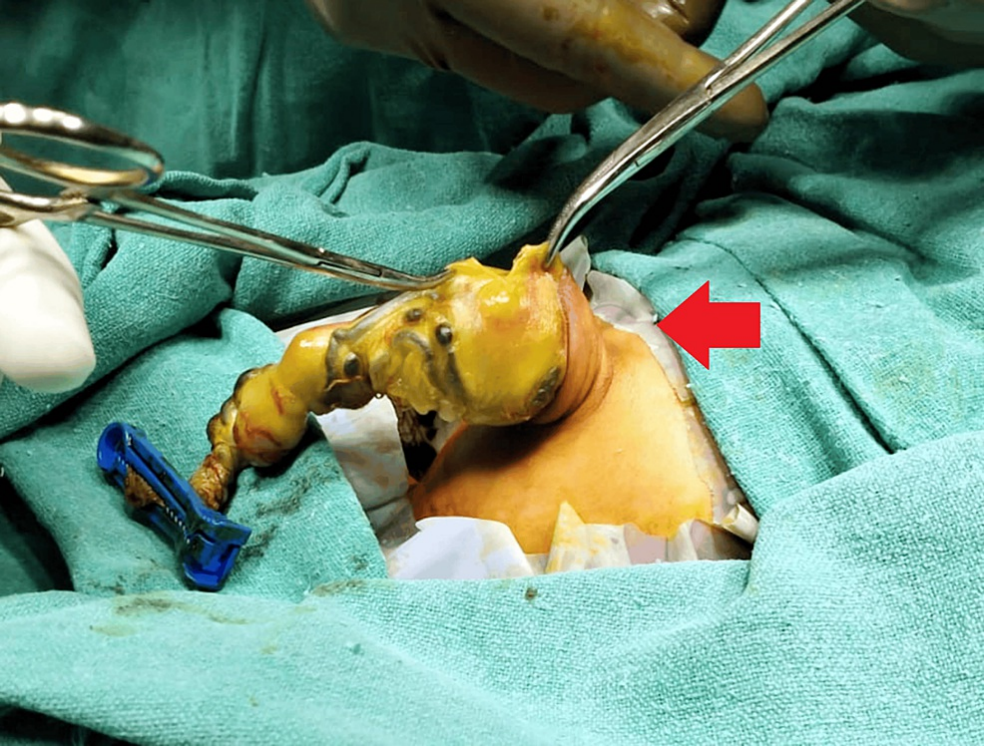
Preoperative image showing omphalocele with herniation of the umbilical cord (red arrow)

An erect abdomen X-ray revealed signs suggestive of intestinal obstruction, prompting emergency surgical intervention (Figure 1). Exploratory laparotomy was performed under general anesthesia, during which the omphalocele sac was meticulously reduced. However, attempts to reduce the bowel contents through the umbilical defect were unsuccessful (Figure 2). The infant was operated on the third day of life. No organ anomalies were detected on 2D- echo and ultrasound of the abdomen. Omphalocele was not detected on prenatal ultrasound.

**Figure 3:**
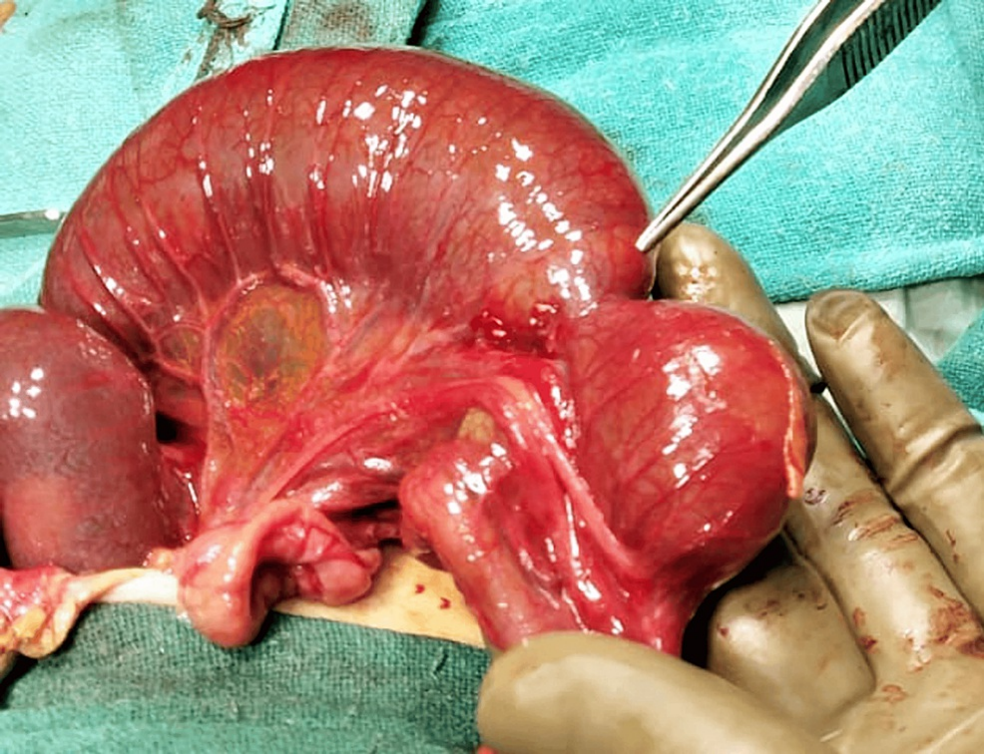
Ileal atretic segment with dilated proximal loop

**Figure 4:**
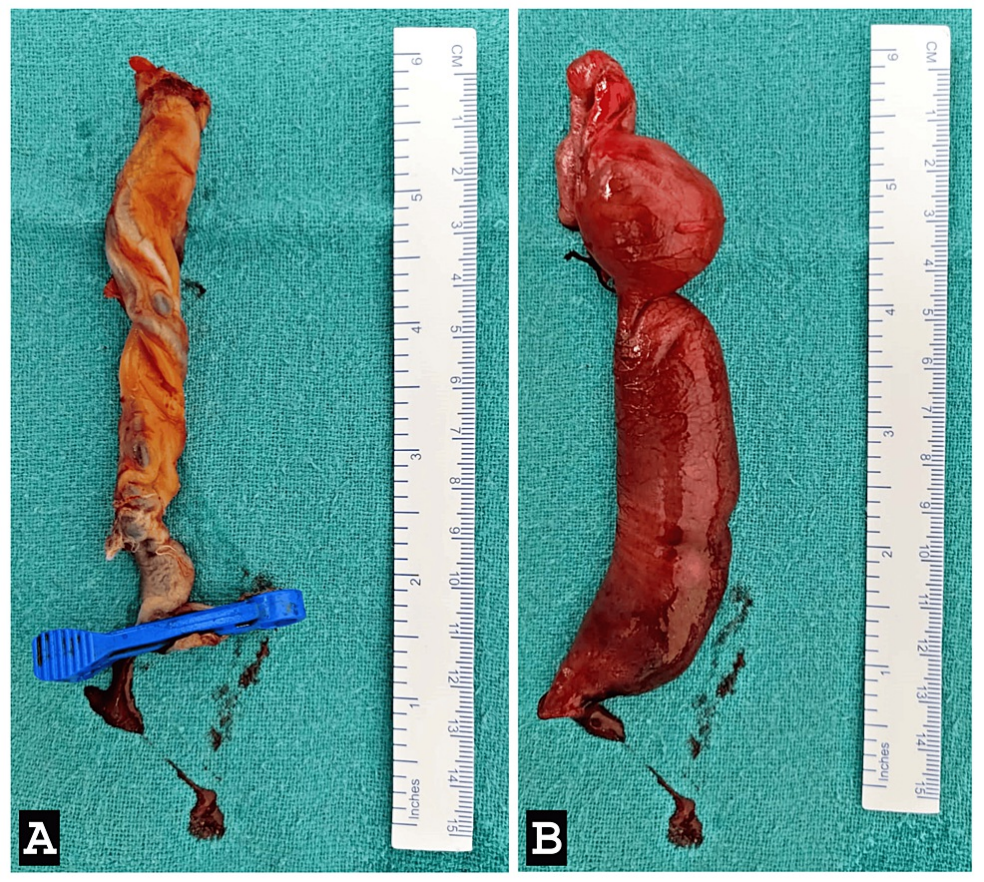
(A) Excised specimen of the umbilical cord with a sac of omphalocele. (B) Resected specimen of ileal atretic segment

**Figure 5:**
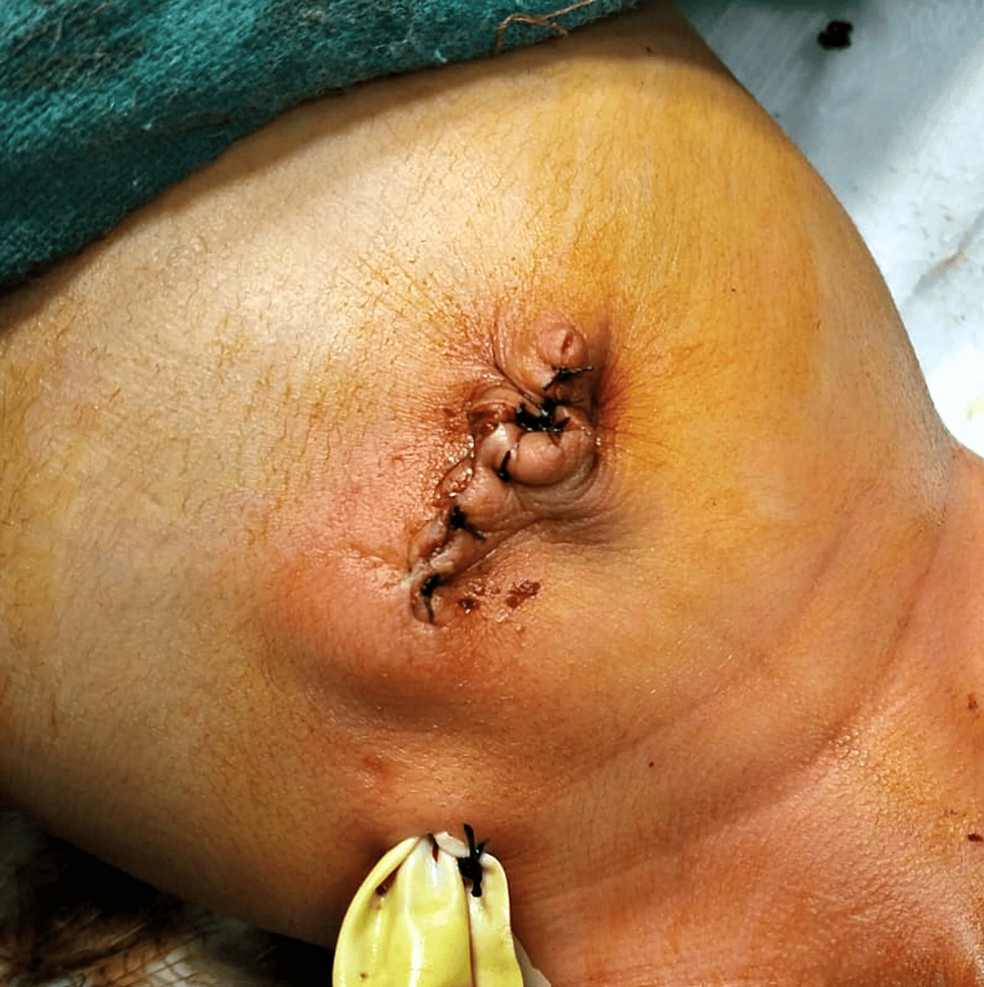
Postoperative suture line with gloved drain

A transverse incision was made at the umbilical level, facilitating a thorough bowel examination. A fibrotic band causing ileal atresia in the proximal ileum was identified (Figure 3). Resection of a segment of grossly dilated redundant ileum, approximately 15 cm in length, including the atretic segment, was performed. Anastomosis was carried out between the proximal and distal ends of the resected ileum (Figure 4). The redundant omphalocele sac was excised, and a glove drain was placed in the right iliac region for drainage purposes (Figure 5). The operative time was two hours and fifteen minutes, and the infant required ventilatory support postoperatively.

Hemostasis was achieved, and the abdominal wall was meticulously repaired. Omphaloplasty and umbilicoplasty were conducted to reconstruct the umbilicus. Careful attention was given to instrument and mop counts to ensure no items were inadvertently left behind. The procedure was completed without complications, and the infant was subsequently transferred to the neonatal intensive care unit for postoperative monitoring and management. The infant succumbed to sepsis on postoperative day 9.

## Discussion

The presented case underscored the intricate management required for neonates with complex congenital anomalies, specifically omphalocele with concurrent ileal atresia. Omphalocele, although rare, is associated with many other congenital disabilities, with gastrointestinal anomalies being among the most common [8]. In this case, the coexistence of omphalocele and ileal atresia posed significant challenges in surgical management. Early recognition and prompt intervention are paramount in cases of omphalocele to minimize the risk of associated complications such as infection, bowel obstruction, and respiratory compromise [9]. In our case, the infant was promptly referred for surgical management following diagnosis at birth, highlighting the importance of timely intervention in improving outcomes.

The concurrent presence of ileal atresia added complexity to the surgical approach. Intestinal atresia, characterized by a narrowing or complete absence of a portion of the intestine, requires resection of the atretic segment and restoration of intestinal continuity through anastomosis [10]. Our surgical approach involved meticulous exploration and resection of the atretic segment, followed by anastomosis of the remaining healthy bowel. This intervention aimed to alleviate the obstruction and restore intestinal function, thereby mitigating the risk of complications such as bowel necrosis and sepsis. Multidisciplinary collaboration is essential in the management of complex congenital anomalies. In our case, neonatologists, pediatric surgeons, and anesthesiologists worked together to ensure comprehensive preoperative evaluation, intraoperative management, and postoperative care. Such collaboration facilitates optimal decision-making and enhances patient outcomes [10]. Furthermore, meticulous attention to detail during surgery is crucial to minimize the risk of postoperative complications. Careful hemostasis, thorough abdominal cavity exploration, and meticulous closure of the abdominal wall are imperative to prevent intra-abdominal sepsis and wound complications [11].

## Conclusions

In conclusion, the successful surgical management of omphalocele with concurrent ileal atresia in the presented case exemplified the importance of a multidisciplinary approach, timely intervention, and meticulous surgical technique in optimizing outcomes for neonates with complex congenital anomalies. The collaborative efforts of neonatologists, pediatric surgeons, and ancillary healthcare professionals facilitated comprehensive preoperative evaluation, intraoperative decision-making, and postoperative care. By addressing the omphalocele and ileal atresia simultaneously, we aimed to minimize the risk of complications such as bowel obstruction and sepsis, thereby improving the long-term prognosis for the patient. Continued research and clinical collaboration are essential to refine surgical techniques further and enhance future management of similar cases.
